# Transient peak-strain matching partially recovers the age-impaired mechanoadaptive cortical bone response

**DOI:** 10.1038/s41598-018-25084-6

**Published:** 2018-04-27

**Authors:** Behzad Javaheri, Alessandra Carriero, Maria Wood, Roberto De Souza, Peter D. Lee, Sandra Shefelbine, Andrew A. Pitsillides

**Affiliations:** 1 Skeletal Biology Group, Comparative Biomedical Sciences, The Royal Veterinary College, Royal College Street, London, NW1 0TU UK; 2 The City College of New York, Department of Biomedical Engineering, 160 Convent Avenue, New York, NY 10031 USA; 3 Universidade Federal de Mato Grosso (UFMT), Departamento de Clínica, Av. Fernando Corrêa da Costa, 2367 - Boa Esperança, Cuiabá, 78060-900 Brazil; 4 Manchester X-Ray Imaging Facility, University of Manchester, Research Complex at Harwell, RAL, Didcot, OX11 0FA UK; 5 Department of Mechanical and Industrial Engineering, Northeastern University, 360 Huntington Ave, Boston, MA 02115 USA

## Abstract

Mechanoadaptation maintains bone mass and architecture; its failure underlies age-related decline in bone strength. It is unclear whether this is due to failure of osteocytes to sense strain, osteoblasts to form bone or insufficient mechanical stimulus. Mechanoadaptation can be restored to aged bone by surgical neurectomy, suggesting that changes in loading history can rescue mechanoadaptation. We use non-biased, whole-bone tibial analyses, along with characterisation of surface strains and ensuing mechanoadaptive responses in mice at a range of ages, to explore whether sufficient load magnitude can activate mechanoadaptation in aged bone. We find that younger mice adapt when imposed strains are lower than in mature and aged bone. Intriguingly, imposition of short-term, high magnitude loading effectively primes cortical but not trabecular bone of aged mice to respond. This response was regionally-matched to highest strains measured by digital image correlation and to osteocytic mechanoactivation. These data indicate that aged bone’s loading response can be partially recovered, non-invasively by transient, focal high strain regions. Our results indicate that old murine bone does respond to load when the loading is of sufficient magnitude, and bones’ age-related adaptation failure may be due to insufficient mechanical stimulus to trigger mechanoadaptation.

## Introduction

Ageing of the skeleton is closely linked to osteoporotic fragility fractures and a failure to retain bone mass. However, the exact mechanisms accounting for this age-related decline in mass that underpin bone’s loss of functional competence have yet to be fully elucidated. Mechanoadaptation is a primary determinant in the maintenance of bone mass, as well as architecture, which ensures sufficient bone strength to withstand external mechanical stimuli without fracture. Numerous studies have investigated the relationship between mechanical loading and bone structure in human (particularly in high impact sports or bedrest^1–3^) and animal bone (with externally applied load or unloading^4–11^), and *in vitro* (applying load or fluid flow to bone cells^12,13^). These studies have established that the mechanoadaptive response depends on magnitude, frequency and rate of loading, and is likely coordinated regionally by osteocytes^9^. It has been shown that the response to loading is more effective in young than in aged bone^14–16^ and therefore, a restricted capacity for adaptation to mechanical demands may underpin the failure to retain bone mass during ageing.

Until recently, it has been assumed that mechanoadaptive responses are progressively and irreversibly diminished with ageing. Based on studies showing that load responses could be boosted by prior sciatic neurectomy (SN) in young mice^17^, it was found that reduced capacity for load-induced osteogenesis in aged mice could indeed be reversed by a short 4 day-long period of SN^18^. These authors speculated that short-term disuse eliminated any age-related differences in the sensitivity to external mechanical stimuli in both cortical and trabecular bone^18^. This increase in sensitivity in both cortical and trabecular bone types was, however, not fully supported by a more recent study which reported that longer-term SN-related disuse more effectively eliminated differences in sensitivity to external mechanical stimuli in aged cortical bone, but instead abolished the beneficial effects of short-term SN in trabecular bone^19^. The origins of such priming events that facilitate a restoration in bones’ mechanoadaptive response to loading in the aged skeleton are incompletely defined and, indeed, whether they might be instigated by alternative short-term modifications in the mechanical environment remains unexplored. Clues to the precise mechanical environments that may serve such priming function in aged bone may emerge from an understanding of which mechanical stimuli engender greatest response to loading in young individuals. In humans, high impact loading [running (3.9–9.2 g) and jumping (5.4–9.2 g)^20^] has been positively related to femoral neck bone mineral density (BMD)^21^, whereas moderate [2.1–4.2 g^20^] or low impact loads [0.5–2.1 g^21^] do not elicit sufficient strain stimulus to induce a mechanoadaptive bone remodelling response. This suggests that only high loads increase BMD whereas low magnitude activity would appear to be of little benefit and, that high magnitude loading which engenders high strains may be the most effective driver of bones’ mechanoadaptive response. This may also be consistent with the long-held view proposed by Burr and colleagues that load-induced microdamage can be significant driver of intracortical bone remodelling^22,23^ which evokes mechanoadaptive changes in bone structure ensuring sufficient strength to prevent fracture^24^.

Peak or maximum dynamic strains of 2,000–3,200 µε have been recorded during habitual use in limb bones of many species^25–27^. It has also been found that loads which exceed these strain magnitudes are sufficient to drive the osteogenic response to mechanical loading^28^. Application of external loading to murine tibia in previous studies have established that loads of 11–13 N applied to mouse tibiae at a range of ages, up to skeletal maturity, promoted mechanoadaptation^14,17,29–31^. In these studies, “osteogenic” strains were typically calibrated by strain gauges and resulted in 2000–2200 με in mature animals^14,29,30^ and lower strains of 1200–1500 με in young growing animals^17,31^. In contrast, other strain gauge studies found that 9 N loads that engendered similar strain levels in aged mice were not sufficient to promote new bone formation^16,32^. It has more recently become clear from strain-mapping by digital image correlation in this same model, that load application does not produce homogenous strains across the tibia; loading instead generates focal regions of low and high strains, which are appropriately neutralised by regionalised mechanoadaptive bone formation^33^. Whilst we do not currently know how the local mechanical environment correlates with regions of bone formation, these observations lead to two questions: (a) does adaptation in aged mice fail because the mechanical stimulus is maintained below a certain threshold and, (b) can this threshold be exceeded to prime mechanoadaptive new bone formation in response to levels of strain to which aged mice would otherwise be insensitive?

Herein we explore if mechanoadaptive responses can be primed in both cortical and trabecular bone of aged female mice, through the imposition of a brief high magnitude load-priming regime. We find that only two bouts of additional high magnitude load, producing maximum strains of ~5500 µε, can effectively prime cortical but not trabecular bone of aged mice to subsequent loading, which engenders strains matching those to which the bone was previously unresponsive. This load-priming of cortical bone of aged mice was regionally correlated with down-regulation of osteocyte SCLEROSTIN expression, used as a proxy measure of osteocyte response to applied load and of regional coordination of mechanically-induced new bone formation. Our data suggest that age-related failure of mechanoadaptation is due to insufficient mechanical stimulus to trigger adaptation and that aged bone’s response to loading can be reactivated by short bursts of high magnitude loading.

## Results

### An excess strain load-priming regime restores cortical bone’s mechanoadaptive response in aged mice

**Figure 1:**
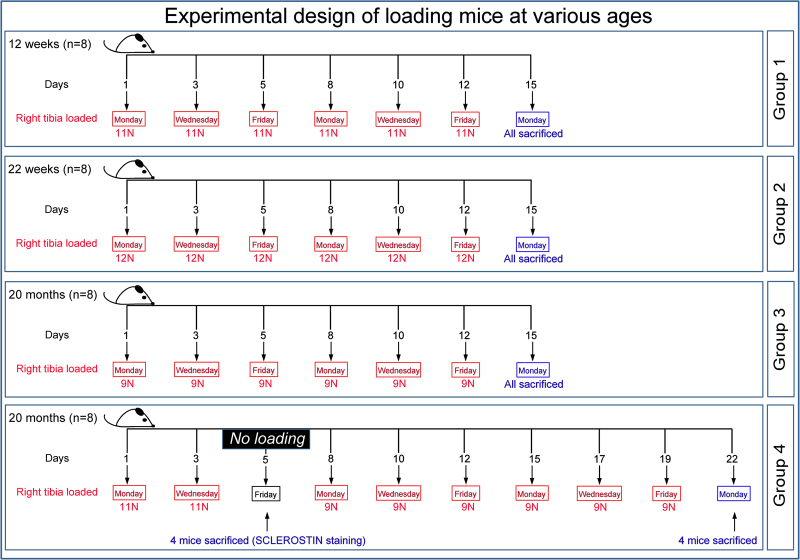
Experimental loading, sacrifice and tissue collection design in mice at various ages. Group 1: 12 week-old loaded at 11 N; group 2: 22 week-old loaded at 12 N; group 3: 20 month loaded at 9 N and group 4: 20 months old subjected to two episodes of 11 N followed by two weeks of 9 N loading (as in group 3). Group sizes were n = 8 for groups 1–3 and 4 for group 4 respectively.

**Figure 2:**
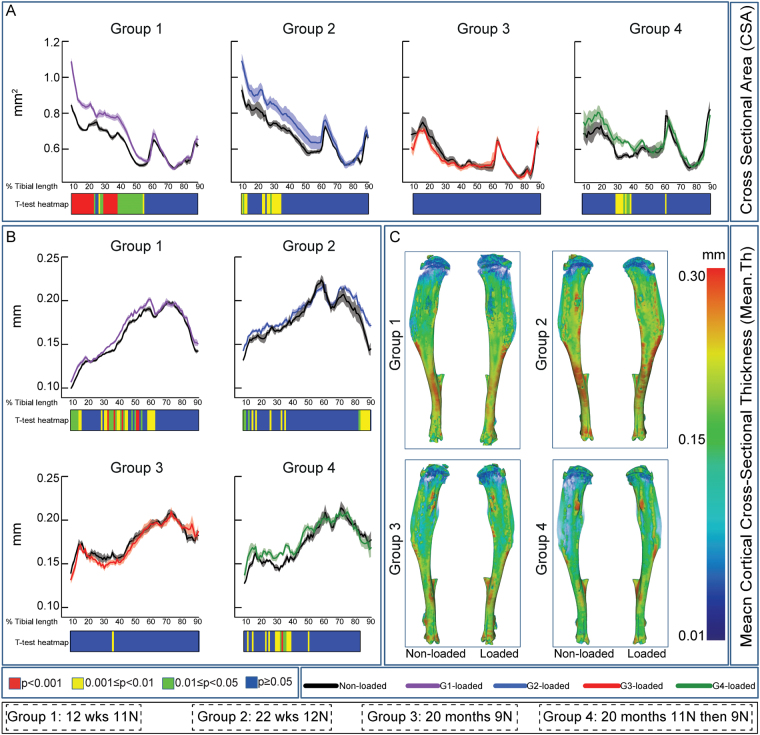
Analysis of cross sectional area (CSA) and mean cortical thickness along the entire tibia length. (**A**) Mean CSA of control and loaded tibiae in female C57/Bl6 at 12 and 22 weeks as well as 20 months of age. (**B**) Mean cortical thickness of control and loaded tibiae of female C57/Bl6 at 12 and 22 weeks as well as 20 months of age. (**C**) Representative 3D micro-CT colour-coded images of tibia cortical bone thickness. Statistical significance of differences in CSA and cortical thickness between control and loaded tibiae along the entire tibia shaft, represented as a heat map. Red p < 0.001, yellow 0.001 ≤ p < 0.01, green 0.01 ≤ p < 0.05 and blue p ≥ 0.05. Group sizes were n = 8 for groups1–3 and 4 for group 4 respectively. Group 1: 12 week-old loaded at 11 N; group 2: 22 week-old loaded at 12 N; group 3: 20 month loaded at 9 N and group 4: 20 months old subjected to two episodes of 11 N followed by two weeks of 9 N loading (as in group 3). Line graphs represent means ± SEM.

We first characterised the tibial mechanoadaptive response to loading at a range of ages and examined whether prior exposure to strain levels, surplus to those predicted by earlier strain gauge studies, was capable of priming mechanoadaptation in aged mice. Based on previous studies^14,17,29–31^ we therefore subjected growing (12 week-old: group 1), skeletally mature (22 week-old: group 2) and aged (20 month-old: group 3) mice to two weeks of dynamic loading at magnitudes of 11, 12 and 9 N, respectively and compared the regionalised mechanoadaptive changes in bone mass and architecture to those induced in another group of aged mice (20 month-old; group 4) subjected initially to two 11 N ‘high loading’ episodes and then either loaded identically to group 3, for 2 weeks at 9 N, or sacrificed 48 hours later (n = 4) for tissue collection (Fig. 1). Whole tibia cortical bone analysis (excluding first/last 10% of total length^9,34,35^) showed significant load-induced increases in cross-sectional area (CSA) predominantly in the proximal region, compared to non-loaded tibiae in both young (group 1) and skeletally mature (group 2) mice; between ~10–55% of tibia length in group 1 and to a similar but lesser extent in group 2 (~10–35%); neither of these showed any significant modification distally (Fig. 2A). Our data show, in agreement with previous findings, that 9 N did not lead to a significant increase of tibia CSA in aged mice (group 3; Fig. 2A). In contrast, the application of two additional bouts of 11 N load priming resulted in significant increases in CSA in proximal regions (30–40%; Fig. 2A).

In addition to CSA, mean cortical cross-sectional thickness was also significantly increased predominantly in the proximal region in the loaded tibia of both growing (group 1 to ~60%) and skeletally mature mice (group 2, to ~35% as well as at 80–90% distally; Fig. 2B,C) compared to control. Consistent with an impaired mechanoadaptive response in aged bone, 20 month-old mice (group 3) showed negligible change in cross-sectional thickness in response to loading (Fig. 2B,C) whilst, in agreement with CSA data, high-load priming reactivated significant mechanoadaptive response in cross-sectional thickness at many predominantly proximal tibial locations in similarly aged mice (10–50% group 4; Fig. 2B,C).

To provide an estimate of tibial resistance to bending forces we also calculated second moment of area around minor (I_min_) and major axes (I_max_) in loaded and non-loaded tibia in all groups. Our data reveal that both I_min_ and I_max_ are significantly increased by load application at many tibia locations in young mice (group 1; ~10–45% and ~10–40%, respectively; Supplementary Fig. 6A,B) but not in skeletally mature (group 2) or aged mice, either without (group 3) or after application of the high-load priming regime (group 4; Supplementary Fig. 6A,B). Predicted resistance to torsion (J) showed almost identical age-related trends and, after high load-priming, no change in the sensitivity to load in tibiae of aged mice (Supplementary Fig. 7B). In contrast, sensitivity to load-induced changes in tibia ellipticity were apparent only in young mice (group 1) and after two bouts of 11 N high load-priming in aged mice (group 4), but not in non-primed aged (group 3) or skeletally mature (group 2) mice (Supplementary Fig. 7A). These data reveal that cortical loading responses are most pronounced during growth, are reduced upon maturation and absent in ageing, when they can be partly restored by a ‘high load’ priming episode.

### High magnitude 11 N load-priming regime does not restore mechanoadaptive response in trabecular bone of aged mice

**Figure 3:**
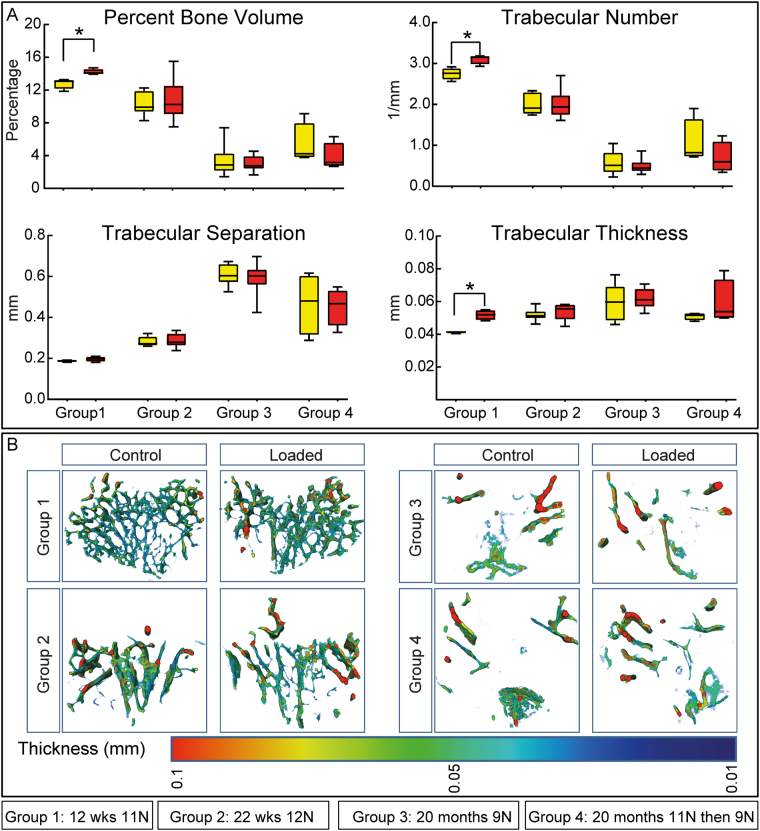
Trabecular bone phenotype in control and loaded tibiae in 10 and 22 week-old, and 20 month-old mice. (**A**) Percent bone volume, trabecular number, separation and thickness. (**B**) Trabecular thickness heatmap of control and loaded tibiae at various ages. Group sizes were n = 8 for groups1–3 and 4 for group 4 respectively. Group 1: 12 week-old loaded at 11 N; group 2: 22 week-old loaded at 12 N; group 3: 20 month loaded at 9 N and group 4: 20 months old subjected to two episodes of 11 N followed by two weeks of 9 N loading (as in group 3). Box-plots represent means ± SEM. *denotes p ≤ 0.05.

Previous studies have disclosed divergent behaviour of bone in trabecular and cortical compartments^18,19^. To determine whether ‘high load’ priming also produces restoration of aged trabecular bone’s loading response we compared trabecular mass and architecture in left (non-loaded) and right (loaded) tibiae in mice from each of the four groups. We found that load application provoked significant increases in percent bone volume, trabecular number and thickness, but not trabecular separation, in young mice (group 1; Fig. 3A,B). No significant load-related increases were however apparent in tibia in skeletally-mature or aged mice, either without (group 3) or with priming at high loads (groups 2–4; Fig. 3A,B). These data reveal that the trabecular bone response to loading is most marked during growth, absent in skeletally mature and aged mice, and that pre-exposure to short bursts of surplus levels of strain fails to exert any modification in trabecular sensitivity to load.

### Load-priming of aged tibia restores osteogenic response in regions of greatest strain-related mechanical stimulus that map to modified osteocyte SCLEROSTIN expression

**Figure 4:**
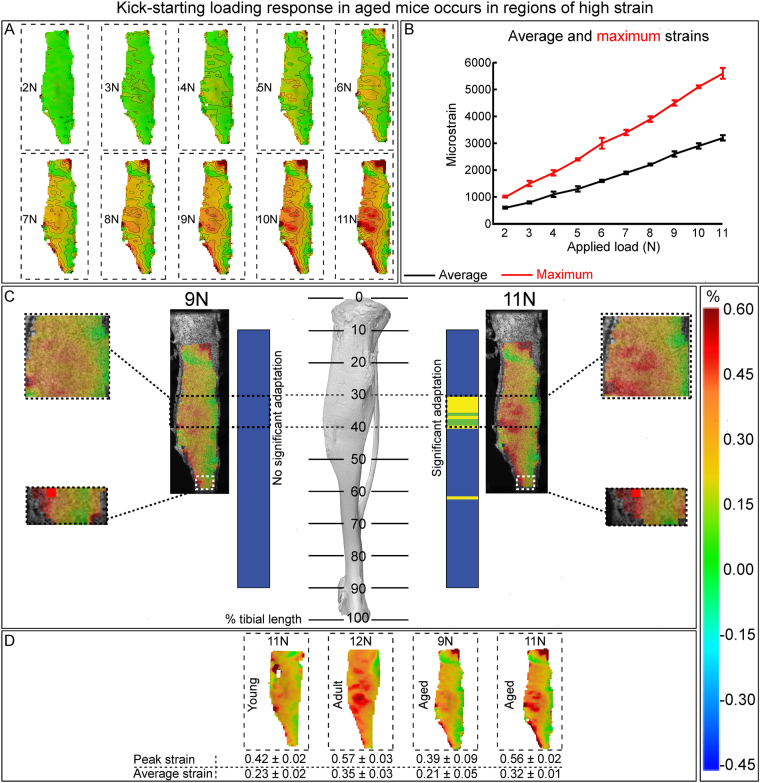
Load-priming of aged tibia restores osteogenic response in regions of greatest strain-related mechanical stimulus. (**A**) *Ex-vivo* strain contour maps demonstrating strain gradient at 2–11 N produced by digital image correlation (DIC) on the medial side of the tibia isolated from 20 months old female C57/Bl6 mice. (**B**) Maximum and average strains at these 10 loading instances. (**C**) Representative strain contour plots on the medial surfaces of the tibiae under 9 N and 11 N axial compression superimposed on the surface of tibia. (**D**) Representative strain contour plots on the medial surfaces of the tibiae under axial compression from young (11 N), mature (12 N) and aged (9 and 11 N) mice. Line graphs represent means ± SEM. Group sizes were n = 4.

To define the mechanical environment providing the regional origins of this high load-priming event, we used digital image correlation (DIC) to measure strains engendered across the entire medial tibia surface by incremental increases in applied loads (up to 11 N) in 20 month-old mice. As transverse and shear strains were both negligible in comparison, Fig. 4 shows only axial strain. In agreement with previous studies^33,34^, compressive loads generated a non-uniform strain field across the curved tibia surface, with tension predominating medially. Application of increasing load correspondingly elevated both average and maximum strain linearly across the tibia surface in aged mice (Fig. 4A,B). Examination of surface strain changes between 9 and 11 N in tibial regions, where high load-priming either restored or failed to restore the mechanoadaptive response (at 30–40% and ~60%, respectively), showed that loading generates focal regions of high strain in those regions where the mechanoadaptive response was reactivated by pre-exposure to 11 N loading (Fig. 4C). Further exploration of surface strains by DIC showed that 11 N load in young mice produced average/maximum strains of 2300/4200 με, that 12 Ν in mature mice produced average/maximum strains of 3500/5700 με (Fig. 4D). In tibiae from 20 month-old aged mice, 9 N loads produced average/maximum strain of 2100/3900 με, similar to those engendered by 11 N in the young mice, whilst increased loads to 11 N reached similar average/maximum strains (3200/5600 με) to those generated in mature mice (by 12 N loads, Fig. 4D). Taken together these data show that: (a) maximum strain appears to determine the load-response; (b) the threshold for maximum strain increases with age and; (c) only once this threshold is reached mechanoadaptation is restored.

**Figure 5:**
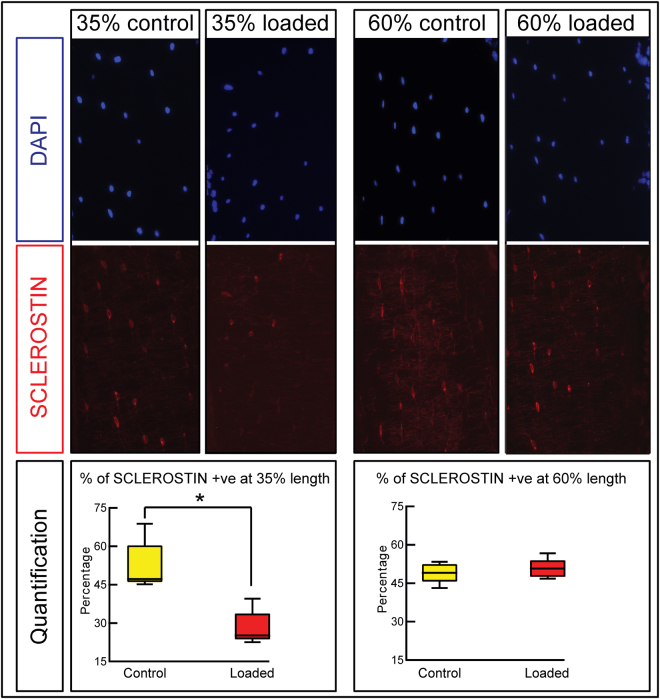
Effect of excess strain load-priming on osteocytes’ SCLEROSTIN expression at 35 and 60% tibia length. 20 months old female mice in group 4 received two bouts of 11 N loading and sacrificed two days thereafter. Representative transverse DAPI and SCLEROSTIN-immunostained images with the percentage changes of SCLEROSTIN-positive osteocytes in non-loaded contralateral control and loaded right tibia demonstrating significant reduction in percentage of SCLEROSTIN-positive osteocytes at 35% of tibia length only. 8 sections from 4 mice per group were quantified. Box-plots represent means ± SEM. Group sizes were n = 4. *denotes p < 0.05.

It has been found previously that osteocytes show reduced SCLEROSTIN expression levels in response to loading and this was therefore used as a proxy marker for osteocyte responsiveness to load^36^. We assessed SCLEROSTIN expression, in a region of highest strain and a distal region where strain was lower and load-response was not significant, in loaded and non-loaded tibiae of aged mice following the two bouts of 11 N load-priming. We found that the percentage of total SCLEROSTIN positive osteocytes was significantly reduced in loaded compared to non-loaded tibia at 35% length (46% reduction; P < 0.01). In contrast, no significant modification in SCLEROSTIN positive osteocyte numbers was observed at ~60% of tibia length (Fig. 5). Together, these data reveal that the sensitivity of osteocytes to loading, assessed by reduction in SCLEROSTIN expression, was restored in the cortex of aged bone in a region of the tibia length where high loads effectively prime the mechanoadaptive new bone formation response to levels of strain to which aged mice were otherwise insensitive.

## Discussion

Our analysis of the entire tibia finds that short-term priming in aged mice with loads high enough to generate maximum strains matching those capable of driving osteogenesis in younger mice, termed surplus high-load herein, can partially restore cortical but not trabecular mechanoadaptive response in aged bone. We confirm previous studies describing a failure of mechanoadaptation in specific cortical regions and extend them by showing that they are absent along the entire length of the aged tibia cortex. By characterising tibial responses to applied load across a range of ages, we show that transient peak-strain matching partially recovers mechanoadaptive responses in aged bone. Intriguingly, this reactivation of mechanoadaptation in aged cortical bone is found where strains are greatest and selective reduction in osteocyte SCLEROSTIN expression is also observed. Previous studies have reported that short and long-term SN-induced disuse also reactivates mechanoadaptive responses in aged mice^18,19^. We find that short bursts of loading, high enough in magnitude to generate strains exceeding those previously considered sufficient to drive osteogenesis in young mice (3,200 µε) also effectively prime greater sensitivity to subsequent loading episodes within this range in the bones of aged mice suggesting that aged bone fails to respond due only to insufficient mechanical stimulus.

The extent of the mechanoadaptive response (and likely its absence) is subject to many variables in the loading environment including the load magnitude, frequency, rate, duration and inclusion of rest insertion^28,37–43^. Our study was focused on manipulating magnitude together with duration by simple inclusion of additional loading episodes. Whilst in our studies a significant mechanoadaptation in young and adult mice is observed in response to 40 cycles/day for 6 days over a two weeks period, these responses are however absent in aged mice. We find, however, that two additional, preceding high load bouts evoke a response that would otherwise be absent, or effectively ‘prime’ the adaptive response. Our study design means that we are unable to apportion this partial recovery to either the additional time or the additional high magnitude bouts of loading with any certainty. Our results nonetheless imply that either or both are required in order for the mechanoadaptive response to be realised in aged bone.

The complete lack of response in aged mice to loading in our studies does not agree with some previous studies^14,16,32,44^. For example, Meakin *et al*., (2014) reported that aged male mice have a different threshold, whereas female mice become less sensitive to loading with ageing^14^. Our studies differ as we report that the adaptation engendered by 6 bouts of low strain loading is observed in the bones of aged mice, only when these are preceded by the insertion of a two, higher load, strain-matched episodes. We also appreciate that different mouse tibial loading studies have employed different loading regimes^15,16,29,37–40,44,45^; which are modifications from the conditions reported in the original study^17^. These inherent differences may make direct comparisons between studies difficult as they could influence our interpretation of different aspect of mechanoadaptation. In our studies we have applied dynamic loading dynamic, 2 N preload, 40 cycles/day, 2 Hz, with 10 s rest periods between cycles on alternate days for two weeks; 6 loading episodes in total. Other studies^15,16,44^ have applied more than 5–30 times higher cycles/day, higher frequency, lower preload, shorter rest period as well as 4 additional loading episodes. Birkhold *et al*., (2014) and Razi *et al*., (2015) applied 216 cycles/day 4 Hz, with 0.1 sec rest period/cycle, for two weeks; 10 episodes in total. Whereas, Holguin *et al*., (2014) went as high as 1200 cycles/day (4 Hz, with 0.1 sec rest periods for two weeks; 10 loading episodes in total).

It is unclear whether aged bones partially or completely lose their mechanical responsiveness due to failure of the osteocytes to sense load, a failure of osteoblasts to lay down new bone, or insufficient mechanical stimulus to trigger a response. By combining DIC tibia strain mapping in aged mice with measurements of whole cortical response to loading we have been able to establish a regional relationship between the periosteal mechanical stimulus and the ensuing spatially-regulated load response along the tibial length, suggesting that aged bone’s responsiveness to loading is simply due to an insufficiency in the mechanical stimulus required to trigger a response. We show that with sufficient stimulus, even when applied a few times transiently, aged bone responds to mechanical load. Our measurements of strain across the tibia’s medial surface by DIC allows for comparison between maximum and average strain, which both increased linearly in line with previous data indicating that we are operating below the damage threshold^17^.

Using our loading model along with analysis of responses in a few selected regions - not entire tibia - we previously reported that loading induces significant magnitude-related increases in bone accrual in growing mice which become somewhat diminished (still present) in mature mice^17^. We now show, using non-biased whole-bone cortex analyses that load-responses are indeed completely lacking along the entire tibia of aged mice; information not otherwise evident in positional analysis of selected regions^34,46^. Several studies by us and others have shown that these responses are lacking^19,32,47^. Herein, we show that prior strain-gauge calibration appears to have significantly underestimated peak strains. We have also matched the location of bone formation with the local stimulus in previous studies^48,49^. Indeed, the majority of the adaptation occurs on the posterior-lateral surface. We find by DIC that threshold peak strains required to elicit a response is increased with ageing and, when DIC is used to match peak strains in aged mice, that an adaptive response is indeed achieved. It is perhaps not surprisingly that the average tibial surface strain at 9 N in aged mice (~2100 με) by DIC is similar to those values reported using strain gauges^14,30^. These previous strain gauge-based studies performed on mature tibiae estimated 8 N (20 week old)^47^, 11.5 N (26 week old^29^, 20 week old^30^) and 12 N (16 week old^14^) to produce 2000–2200 με. Our average strain by DIC in 22 week old mice at 12 N is 3500 με, which is significantly higher than estimated by strain gauges. This is likely due to the typical location of strain gauge application not matching the region of peak strains more effectively revealed by the extensive mapping of strain by DIC; we have previously shown that the location of the gauge significantly alters the strain measured^33^. When strains are fairly homogenous across the tibia (10 week at 11 N and 20 month at 9 N shown in this study), the average strain with DIC matches the strain reported by strain gauges. When there are high strain regions, however, gauges underestimate the average tibial strain and significantly underestimate peak strains. This results in ‘calibrated’ loads that are smaller than required to accurately match peak strain magnitudes; as in aged tibia at 9 N.

We and others have previously shown that age-related decline in mechanoadaptation can be restored after 4 or 100 days of disuse achieved through invasive neurectomy^18,19^. Together our data support a view that osteocytic sensing and osteoblastic responses are intact and cortical adaptation can be achieved in the aged either by activating remodelling or by delivering sufficient mechanical stimulus. Perhaps, an explanation for this restoration can be found by closer inspection of DIC data from loaded bones which show that average levels of strain increase less markedly than maximum strains, suggesting that spatial strain gradients across the cortical surface are steeper at greater load magnitude. Thus, higher local strain gradients may provide sufficient stimulus to prime bones’ mechanoadaptive response to levels of strain otherwise below the response threshold. This nevertheless raises questions regarding the sequelae of the transient, high load priming stimulus.

By examining bones collected immediately after the priming stimulus, we find that one consequence is a localised decrease in osteocyte SCLEROSTIN expression, which supports a local sensitivity to the mechanical stimulus and the contribution of osteocytes to mechanoadaptation. It is also reported, however, that extremely high loads can create localised micro-damage^22–24,50^ and, perhaps this provokes remodelling to prime responses to ensuing loads of lower magnitude. Our early studies using histological evaluation of young tibiae have shown that 13 N loading in our system fails to induce microcracks^17^. Despite the possibility that 11 N loads may cause microcracks in old bone and that as proposed by Burr and colleagues that this may evoke mechanoadaptive changes in bone structure, it is difficult to reconcile microdamage as a driver of the recued mechanoadaptive response induced by SN in aged animals^18,19^. It is also interesting to emphasise that clinical data from Routh *et al*., (2005) in which it has been demonstrated that aged human osteoporotic bone in patients with scoliosis retains its adaptive response to the mechanical environment^51^.

The loading regime we have used indeed excludes forces that likely herald mechanical failure (the yield point) and fracture. Indeed, a range of studies have used a variety of methods, including, tensile^52^, torsional^53,54^, four-point bending^54–56^ and three-point bending^57–59^ strategies to determine failure strains in small animal models. Whilst the latter is inherently disadvantaged by creating high shear stress near the midsection of the bone, it has become the preferred method for assessing the mechanical properties of mouse bones^59,60^. Using these methods Willingham *et al*., (2010) and Ng *et al*., (2016) reported yield loads for femur in female mice at 20 months of age at 16.5 and 19 N respectively^61,62^. Moreover, Saless *et al*., (2010) also reported yield load of 14.1 N in 4 month old female mice; other studies have provided data close to this range^63–65^. It is evident therefore that the 11 N load applied to aged mouse bones in our study is much lower than would likely be required to generate any structural damage to the tibia. Whilst we have also previously reported that our axial loading model does not produce microcracks in mice of younger age^17^, we acknowledge that these yield load/stress data nonetheless depend on testing conditions (rate, support structure, loading tip, dry or wet) and therefore microdamage remains a possible contributor to the outcomes we have observed in these aged bones.

To date, it has been impossible to resolve components of the complex mechanical environment in an *in vivo* model system where the architectural outcomes can readily be matched with both the strain environment and local changes in cellular behaviour. Bone adaptation to loading is achieved by both anti-resorptive and pro-osteogenic changes; both are thus targets to control bone mass in the aged. Pharmacological tools to limit bone resorption (primarily bisphosphonates) have been successful, but approaches for increasing osteogenesis to improve quality and fracture resistance have not. Control of osteogenesis needs to be spatially targeted as indiscriminate new bone accrual is likely deleterious^66^. Load-related bone formation is likely tightly regulated^67^, but until now it has not been possible to spatially correlate mechanical stimuli and ensuing osteogenic responses along an entire bone length. Our data suggest that load-induced cortical bone accrual in aged mice is targeted and spatially matched to regions of peak strain. Our findings also indicate, however, that high-load priming fails to reactivate mechanoadaptive responses in trabecular bone. Willie *et al*., (2013) reported that trabecular and cortical bone responses to loading are not always identical^45^ and that they diverge with ageing. The authors suggested that trabecular bone loss with ageing coincides with lower strains and hypothesised that trabecular bone may indeed respond to loading if sufficient load is applied. DIC is only able to measure periosteal strains and trabecular strains are likely lower as trabeculae are closer to the neutral axis. Our data support this diminished trabecular responses with ageing and extend this by showing that our high-load priming regime also fails to elicit a trabecular mechanoadaptive response.

It is generally held that osteocytes are central orchestrators of bone mechanoadaptation and osteocytic Wnt signalling is essential in bones’ regionalised anabolic load response^9^. An age-related diminution in these osteocyte responses has indeed been offered as an explanation for failed mechanoadaptation in aged bone^9,47^. This linkage between Wnt activation and load-induced new bone formation is strengthened by regional correlation between changes in osteocyte SCLEROSTIN expression and osteogenesis^36,68–70^. The intimacy of this linkage allows SCLEROSTIN expression to be used as a proxy measure of osteocyte response to loading and of regional coordination of mechanically-induced new bone formation. We hypothesised that targeted recovery of load-response in aged mice is dependent on high strain and resultant changes in this osteocyte marker should reflect such events. This was supported by measurements of osteocyte SCLEROSTIN expression in a region where load-response matched to peak strain which showed, in agreement with previous studies, that the load-response is accompanied by localised reduction in osteocyte SCLEROSTIN expression^68–70^, demonstrating that osteocytes within aged bone are sensitive to loading when the mechanical stimulus is sufficiently high.

In conclusion, our data indicate that cortical bone mechanoadaptation can be primed noninvasively in aged mice, is dependent on regional distribution and induction of osteogenic strains and that an established osteocyte response to loading, namely SCLEROSTIN expression, regionally correlates with local load-induced bone responses and focal regions of high strain.

## Methods

All data generated or analysed during this study are included in this published article (and its Supplementary Information files).

### Animals

Female C57BL/J6 mice (Charles River Company, UK) were randomized and housed in polypropylene cages in groups of 4, subjected to 12 h light/dark cycle with room temperature at 21 ± 2 °C and fed ad libitum with maintenance diet (Special Diet Services, Witham UK). All procedures were carried out in accordance with the Animals (Scientific Procedures) Act 1986, an Act of Parliament of the United Kingdom and were approved by the Royal Veterinary College Ethical Review Committee and the United Kingdom Government Home Office under specific project licence.

### Load-related tibial bone surface strains using digital image correlation (DIC)

Digital image correlation (DIC) was used as a well-established method to measure strains and to describe strain distribution directly engendered by load application^34^. The cups ensured the bone to be loaded axially across the knee and ankle joints^17^. 10–11 week, 22 week and 20 months-old female C57/Bl6 mice (n = 4) were euthanized, right tibia exposed and covered with a thin layer of matt, water-based, white paint. Bones were subsequently speckled with matt, acrylic, black ink using a high precision air brush^71^. Legs were inserted in custom built loading cups attached to a material testing machine (Instron 5800, High Wycombe, UK) and loaded at a rate of 8 N/min up to 11 N. Two CCD cameras (100 mm lenses, GOM GmbH, Germany) were positioned horizontally in front of the loading cups, at a distance of 42 cm, to provide a 15 × 12 mm field of view with 1.2 mm depth of focus. The two cameras were rotated towards each other meeting at 25° angle on the bone surface. A high-precision 15 mm × 12 mm panel was used for calibration (GOM GmbH, Germany). The bone was illuminated by two diode lamps with polarised filters. During loading, images of the medial side of the tibia surface were recorded at 1 N interval using the ARAMIS 5 M System (GOM GmbH, Germany), with a resolution of 7.5 × 10.9 µm. Post processing of the images was done using 19 × 19 pixel square facets, with 15 pixels step facet. Strains were computed with a computation size of 5 and a validity quote of 65%. Accuracy was determined at zero loading (zero strain) by taking three images in the un-deformed state. Maximum and average strains on the medial surface were calculated from 2–11 N at 1 N interval for all samples. The noise was consistent throughout all tests and of approximately 0.04%.

### *In vivo* loading

Apparatus and protocol for dynamic loading of the mouse tibia/fibula have already been described^17^. Briefly, under isoflurane-induced anaesthesia the right tibia of each mouse was held in the cups and dynamic (40 cycles/day, 2 Hz, with 10 s rest periods between cycles) axial loads were applied through the knee on alternate days for two weeks; 6 loading episodes in total. Based on previous studies^14,16,17,29–32^, employing load: strain calibration by strain gauging, different peak loads for young, mature and aged mice were used, with growing (12 week-old), skeletally mature (22 week-old) and aged (20 month-old) mice subjected to two weeks of dynamic loading at magnitudes of 11, 12 and 9 N, respectively, with another group of aged mice (20 month-old) subjected initially to two 11 N ‘high loading’ episodes and then either loaded identically at 9 N, or sacrificed 48 hours later (n = 4) for tissue collection. The loaded mice were able to move around in the cage and gained access to food and water without difficulties and no adverse effect was observed. Three days after the final loading episode, mice were sacrificed by cervical dislocation; right and left (left acting as contra-lateral control) tibiae were dissected, fixed in 4% formaldehyde (Alfa Aesar Inc., Ward Hill, MA, USA), and stored in 70% EtOH for either scanning or histology.

### X-ray microcomputed tomography (μCT)

Tibia micro-CT scanning and analysis was performed as described^34,46^. Briefly, tibiae were scanned using Skyscan 1172 (Skyscan, Kontich, Belgium) with x-ray tube at 50 kV, 200 µA, 1600 ms exposure time and 5μm voxel size (~2 hours/sample scan time). Slices were reconstructed using NRecon 1.6.9.4, 2D/3D analyses performed using CTAn 1.15.4.0+ version software and CTvox 3.1.0 r1167 version (Skyscan, Kontich, Belgium) used for 3D visualisation of colour-coded images of trabecular thickness.

### Morphometric trabecular bone analysis

Appearance of the trabecular ‘bridge’ connecting the two primary spongiosa bone ‘islands’ was set as a reference point for analysis of proximal tibia metaphyseal trabecular bone; 5% of total bone length from this point (towards diaphysis) was utilised for trabecular analysis.

### Whole bone cortical analysis

Whole bone analysis was performed using BoneJ^72^ (version 1.4.0, an ImageJ^73^ plugin). Following segmentation, alignment and fibula removal from the dataset, a minimum threshold was used in “Slice Geometry” (BoneJ). The main characteristics of bone, measured and reported here, that determine its strength include the quantity of bone material present [mass; cross sectional area (CSA), mean thickness (Ct.Th)] and the distribution of this material in space [shape; second moment of area around the minor (I_min_) and major axes (I_max_), ellipticity and predicted resistance to torsion (J)].

### Immunohistochemistry

SCLEROSTIN staining was performed on mice from group 4 that were sacrificed two days after they experienced two bouts of high load as described previously^14,70^. Briefly, sections (*n = 4*/per mouse from each location along the tibia length analysed) were placed on SuperFrost positive charged (Fisher Scientific) slides. Paraffin was removed by a sequential dip in xylene and rehydrated through graded washes of EtOH and H_2_O and then PBS. Sections were incubated in blocking solution (2.5% BSA, 1% donkey serum in PBS) overnight at 4 °C. The following day, sections were incubated with primary antibody against: SCLEROSTIN (R&D System Minneapolis, MN) at a 1:100 dilution overnight at 4 °C. Sections were washed three times with PBS and incubated for 1 hour with Cy3 conjugated Donkey anti goat (or anti-rabbit) secondary antibody (Jackson ImmunoResearch Laboratories, West Grove, PA) (1:200 in blocking solution) and DAPI. Fluorescent microscopy was performed using a Leica Q550IW florescence microscope with a DC 500 Leica digital camera. SCLEROSTIN positive cells were counted as described previously^14^. Briefly, images of the posterior-lateral regions were taken and positive cells manually counted using ImageJ^73^. The number SCLEROSTIN positive cells expressed as percentage of the total number of osteocytes, as determined by DAPI staining, for both right loaded and left non-loaded control tibiae in 35% and 60% tibial length.

### Statistical analysis

Normality and homogeneity of variance, was used for all comparisons between control and loaded tibiae within the same group. Violations of normality and homogeneity were not observed.

For trabecular bone analysis and SCLEROSTIN immunohistochemistry, graphs were developed using GraphPad Prism 6 (GraphPad Software, Inc., San Diego, CA). For gross cortical bone morphology analysis, graphs were plotted using programming language ‘R’, version 3.1.3 (R Foundation for Statistical Computing, Vienna, Austria; http://www.r-project.org). For this purpose, the functions lattice and grid were used. For all analysis, two-sample t-test was used for comparisons between control and loaded tibiae of the same group. Data are presented as mean ± SEM and considered statistically significant when p < 0.05.

## Electronic supplementary material

Supplementary Information
