# Supplementary material for: Transient peak-strain matching partially recovers the age-impaired mechanoadaptive cortical bone response
Source: Sci Rep. 2018 Apr 27;8:6636. doi: 10.1038/s41598-018-25084-6 (PMC5924380; doi:10.1038/s41598-018-25084-6)
Supplement: Supplementary file 1 — Supplementary Information [file 41598_2018_25084_MOESM1_ESM.pdf]

Transient peak-strain matching partially recovers the age-impaired mechanoadaptive cortical bone response

Behzad Javaheri[1,*], Alessandra Carriero[2], Maria Wood[1], Roberto De Souza[3], Peter D. Lee[4], Sandra Shefelbine[5] and Andrew A. Pitsillides[1]

[1] Skeletal Biology Group, Comparative Biomedical Sciences, The Royal Veterinary College, Royal College Street, London, NW1 0TU, UK

[2] The City College of New York, Department of Biomedical Engineering, 160 Convent Avenue, New York, NY 10031, USA

[3] Universidade Federal de Mato Grosso (UFMT), Departamento de Clínica, Av. Fernando Corrêa da Costa, 2367 - Boa Esperança, Cuiabá, 78060-900, Brazil

[4] Manchester X-Ray Imaging Facility, University of Manchester, Research Complex at Harwell, RAL, Didcot, OX11 0FA, UK

[5] Department of Mechanical and Industrial Engineering, Northeastern University, 360 Huntington Ave, Boston, MA 02115, USA

[*]bjavaheri@rvc.ac.uk

Supplementary information

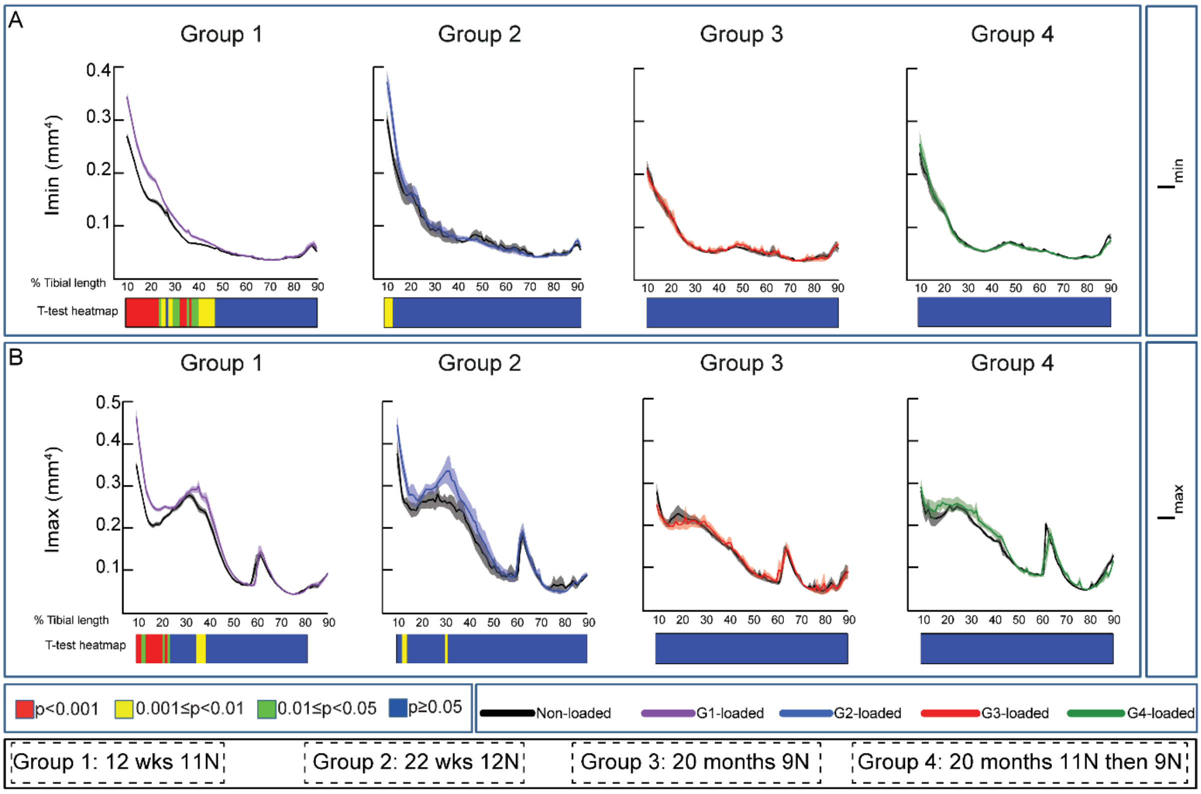

Figure 6. Analysis of I_{min} and I_{max} along the entire length of the tibia. (*A*) Mean I_{min} of control and loaded tibiae of female C57/Bl6 at 12 and 22 weeks as well as 20 months of age. (*B*) Mean I_{max} of control and loaded tibiae of female C57/Bl6 at various ages. Statistical significance of differences in CSA and thickness between control and loaded tibiae along the entire tibial shaft, represented as a heat map. Red $p<0.001$, yellow $0.001\leq p<0.01$, green $0.01\leq p<0.05$ and blue $p \geq 0.05$. Group sizes were n = 8 for groups1-3 and 4 for group 4 respectively. Group 1: 12 week-old loaded at 11N; group 2: 22 week-old loaded at 12N; group 3: 20 month loaded at 9N and group 4: 20 months old subjected to two episodes of 11N followed by two weeks of 9N. Line graphs represent means ± SEM.

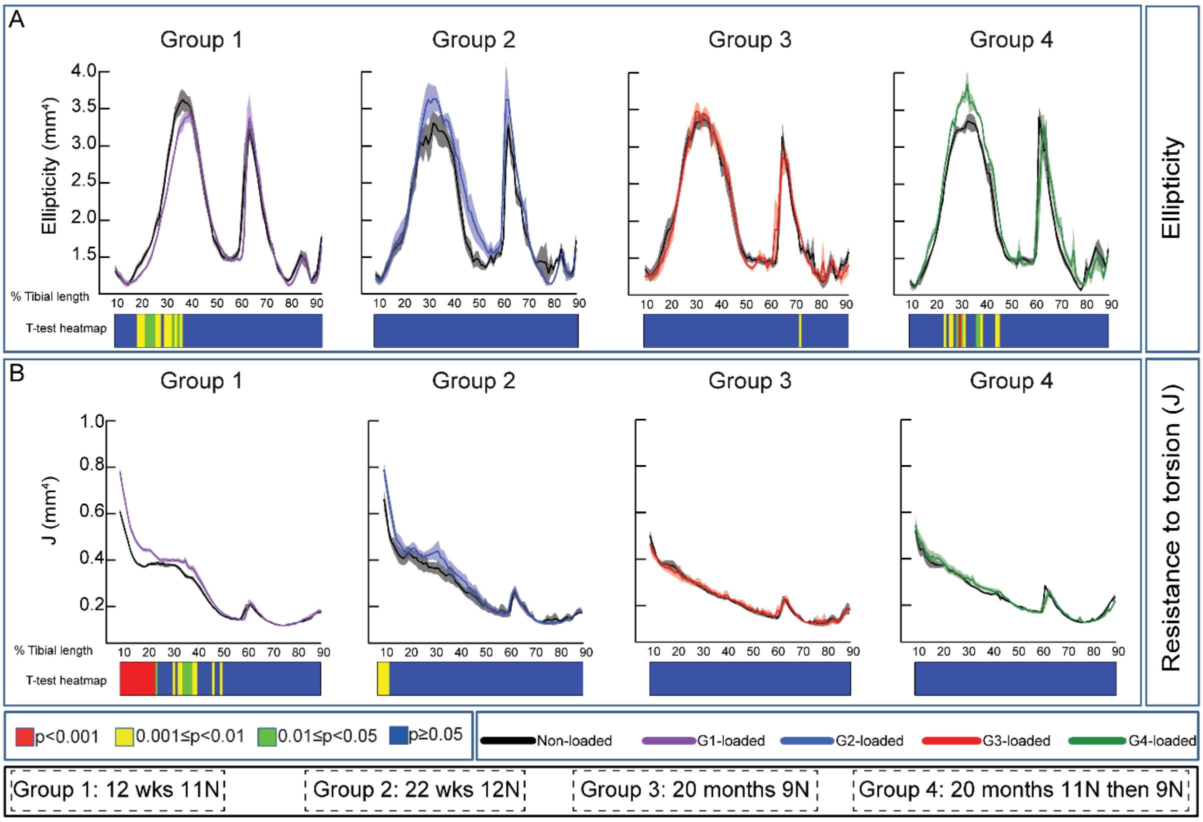

Figure 7. Analysis of ellipticity and predicted resistance to torsion (J) along the entire length of the tibia. (*A*) Ellipticity of control and loaded tibiae of female C57/Bl6 at 12 and 22 weeks as well as 20 months of age. (*B*) Predicted J in control and loaded tibiae of female C57/Bl6 at various ages. Statistical significance of differences in CSA and thickness between control and loaded tibiae along the entire tibial shaft, represented as a heat map. Red $p<0.001$, yellow $0.001 \leq p<0.01$, green $0.01 \leq p<0.05$ and blue $p \geq 0.05$. Group sizes were $n = 8$ for groups1-3 and 4 for group 4 respectively. Group 1: 12 week-old loaded at 11N; group 2: 22 week-old loaded at 12N; group 3: 20 month loaded at 9N and group 4: 20 months old subjected to two episodes of 11N followed by two weeks of 9N. Line graphs represent means ± SEM.
